# Radial line-scans as representative sampling strategy in dried-droplet laser ablation of liquid samples deposited on pre-cut filter paper disks^☆^

**DOI:** 10.1016/j.sab.2014.07.023

**Published:** 2014-11-01

**Authors:** Winfried Nischkauer, Frank Vanhaecke, Sébastien Bernacchi, Christoph Herwig, Andreas Limbeck

**Affiliations:** a Institute of Chemical Technologies and Analytics, Vienna University of Technology, Vienna, Austria; b Department of Analytical Chemistry, Ghent University, Ghent, Belgium; c Institute of Chemical Engineering, Vienna University of Technology, Vienna, Austria

**Keywords:** Laser ablation, ICP-OES, dried-droplet quantification, Biochemical fermentation

## Abstract

Nebulising liquid samples and using the aerosol thus obtained for further analysis is the standard method in many current analytical techniques, also with inductively coupled plasma (ICP)-based devices. With such a set-up, quantification via external calibration is usually straightforward for samples with aqueous or close-to-aqueous matrix composition. However, there is a variety of more complex samples. Such samples can be found in medical, biological, technological and industrial contexts and can range from body fluids, like blood or urine, to fuel additives or fermentation broths. Specialized nebulizer systems or careful digestion and dilution are required to tackle such demanding sample matrices. One alternative approach is to convert the liquid into a dried solid and to use laser ablation for sample introduction. Up to now, this approach required the application of internal standards or matrix-adjusted calibration due to matrix effects. In this contribution, we show a way to circumvent these matrix effects while using simple external calibration for quantification. The principle of representative sampling that we propose uses radial line-scans across the dried residue. This compensates for centro-symmetric inhomogeneities typically observed in dried spots. The effectiveness of the proposed sampling strategy is exemplified via the determination of phosphorus in biochemical fermentation media. However, the universal viability of the presented measurement protocol is postulated. Detection limits using laser ablation-ICP-optical emission spectrometry were in the order of 40 μg mL^− 1^ with a reproducibility of 10 % relative standard deviation (n = 4, concentration = 10 times the quantification limit). The reported sensitivity is fit-for-purpose in the biochemical context described here, but could be improved using ICP-mass spectrometry, if future analytical tasks would require it. Trueness of the proposed method was investigated by cross-validation with conventional liquid measurements, and by analyzing IAEA-153 reference material (Trace Elements in Milk Powder); a good agreement with the certified value for phosphorus was obtained.

## Highlights

• Sample preparation via deposition of liquids on pre-cut filter disks.
• Sample introduction via laser ablation of dried filters.
• Representative sampling avoids bias.
• Signal quantification via external calibration and aqueous standard solutions.
• Alternative approach for quantifying P in challenging matrix.

## 1 Introduction

Controlling nutrients in fermentation media has become a key aspect in process monitoring as biochemical fermentations gain more and more importance in industry and research. Limitations in biomass production caused by a lack of phosphorus are a current focus in biochemical engineering. With a better understanding of this phosphorus-limitation, optimum productivity can be achieved while applying just the required amount of this element. Moreover, the growth-rate of biomass can be controlled via the availability of phosphorus.

Studying phosphorus-limitation requires quantification of total phosphorus, as well as total dissolved phosphorus, during the biochemical reaction (TP and TDP, respectively, as defined in [1]). While conventional approaches using UV–vis spectrometry of the phosphomolybdenum blue complex are highly sensitive for phosphate, they are difficult to implement with the biochemical fermentation matrix at hand because the formation of complexes is interfered by the co-presence of silicate, nitrate, nitrite, sulphide, and several metal ions [1]. Another drawback is that the phosphomolybdenum blue complex is formed exclusively from the phosphate anion. Therefore, UV–vis spectrometry requires full chemical digestion of the samples in order to also detect phosphorus contained in species different from phosphate [1], [2].

A powerful alternative for the established, but cumbersome photometric approach is atomic (emission, absorption or mass) spectrometry, as well as X-ray fluorescence spectrometry (XRF). With these methods, TP and TDP quantification can be achieved without (complete) digestion since these methods are generally not influenced by speciation. Inductively coupled plasma-optical emission spectroscopy (ICP-OES) and mass spectrometry (ICP-MS), as well as atomic absorption spectrometry (AAS) are suitable techniques for this task. We will not expand on AAS further because the method presented in this contribution was designed with the aim of future extension to multi-element quantification. Nevertheless, AAS is a powerful technique for TP single-element quantification [3], also with the option of (limited) multi-element capabilities using continuum sources. XRF and especially total reflection XRF are also possible methods for phosphorus determination. However, sensitivity for elements with low mass number is low and direct analysis of samples without pre-treatment is a difficult task [4], [5].

ICP-OES and ICP-MS are rugged techniques, but analyzing fermentation media without any sample preparation at all remains challenging. One reason for this is the presence of high concentrations of organic compounds and inorganic salts. Such compounds can block sample introduction interfaces and therefore, sample dilution is common practice. In order to facilitate sample preparation and to enhance sample throughput, alternatives for sample dilution are required. Ideally, sample preparation should be straightforward (or automated) with the option of being carried out completely on-site. Moreover, samples should be stabilized to allow for simplified transport, and for archiving. In addition, the method should be tolerant towards suspended particulates (e.g., agglomerates of proteins, cell membranes), especially in the case of TP quantification.

One way to meet these conditions is to prepare dried droplets of the samples. This approach can be performed under field conditions and results in minimum work-load. Elemental analysis of such samples can then be performed later on, using suitable solid-sampling techniques (e.g., laser ablation combined with ICP-MS [6] or ICP-OES, as well as solid sampling graphite furnace AAS [7]).

In this article, we present a measurement protocol for laser ablation, which allows analyzing representative sub-samples of a dried sample droplet. Thus, systematic errors commonly observed with dried-droplet sample preparation caused by inhomogeneous sample distribution on the supporting substrate are effectively overcome. The approach presented here allows for external aqueous calibration, reduces sample-handling to a minimum (no dilution, no addition of internal standard), and thus offers a simplified quantitative method for TDP-quantification in biochemical fermentation media.

## 2 Materials and methods

### 1 Reagents and consumables

High-purity water was prepared in-lab using an Easypure water system (Thermo, USA, resistivity 18.2 MΩ cm). All reagents used were of analytical grade or higher, unless stated otherwise. Concentrated nitric acid, sodium chloride, saccharose and L-tartaric acid were purchased from Merck, Germany. A phosphate stock solution of 10 g L^− 1^ P was prepared by dissolving NaH_2_PO_4_*2H_2_O in high-purity water. This stock solution was quantified versus a P-containing multi-element standard solution (ARISTAR, VWR Germany) using solution-nebulisation ICP-OES analysis to determine its exact concentration. For method validation, IAEA-153 reference material (Trace Elements in Milk Powder) was used. The dried milk-powder was reconstituted by adding high-purity water, shaking and ultrasonication for 30 minutes. Although not identical in sample matrix, this reference material has a similar composition as the samples investigated (i.e., high concentration of organic compounds, high load of Na, Mg, and Ca).

### 2 Instrumentation

Measurements were performed using an iCAP 6500 ICP-OES spectrometer in radial view mode, using a quartz torch and a quartz injector tube of 2 mm inner diameter (Thermo Scientific, USA). Laser ablation (LA) was carried out with a NWR 213 nm solid state Nd:YAG laser (New Wave Research, USA). The ablation chamber was equipped with an ablation cup, which allows for fast wash-out, while offering a large volume ablation chamber. During LA, a gas flow of 0.9 L min^− 1^ of helium was flushed through the cell. After the ablation chamber, a gas flow of 0.4 L min^− 1^ Ar was added via a Y-connector. The LA-system was connected directly to the ICP-torch via 1 m of PTFE tubing of 4 mm inner diameter.

While continuously ablating a spiked filter disk (10 μL of spiked fermentation medium dispensed and dried on pre-cut paper filters), plasma conditions and gas flow rates were initially optimized. The pixel position for all emission lines was checked prior to each measurement session and adjusted to obtain best results in terms of signal-to-noise ratio and to ensure accurate background correction. A summary of the final ICP-OES parameters used for LA, as well as for conventional pneumatic nebulisation (PN, see also Section 5) can be found in Table 1.

**Table 1:** Operating parameters of the ICP-OES Spectrometer.

		LA	PN
Plasma power	W	1450	1400
Radial observation height	mm	15	11
Plasma gas flow rate	L min^− 1^	12	12
Nebulizer gas flow rate	L min^− 1^	0.4^a^	0.7
Auxiliary gas flow rate	L min^− 1^	0.6	0.6
Analytical wavelengths (nm)			
P	177.495^b^		178.284^c^
Au	208.209		-

^a^ make-up gas for LA carrier gas, ^b^ used for quantification, ^c^ used for quality control.

### 3 Elemental mapping and quantification via LA

For LA-ICP-OES mapping of the P-distribution, the dry filters were coated with a thin layer of gold using an Agar B7340 sputter coater (Agar Scientific Limited, Essex, UK). Samples were placed 5 cm away from the gold target, and a sputtering current of 10 mA was applied for 60 s (chamber pressure approx. 0.1 mbar). Later, when ablating these Au-coated samples with the LA system, the omnipresent gold signal (corresponding to all ablation passes) was used to automatically detect individual ablation patterns via ImageLab software (developed by H. Lohninger [8]). With this software, reconstructing the elemental map from the individual laser patterns is significantly facilitated and fully automated. Sample-inherent elements could not be used as tracers for the ablation patterns, as their concentrations either varied laterally (e.g., P, Na), or resulted in detector saturation (carbon). In the context of mapping, only qualitative information was required. Hence, the gold signal was used exclusively for automatic data evaluation, and to identify signal drift.

LA parameters were initially optimized for best signal-to-noise ratio and for fast data acquisition (high laser scan speed to reduce measurement time) by ablating a spiked fermentation sample applied on a pre-cut paper disk. LA maps were recorded by performing adjacent and parallel line-scans (conditions see Table 2). For mapping purposes, signals were recorded in time-resolved mode using readout intervals of 2.5 s.

**Table 2:** Operating parameters of the laser ablation system.

System	NWR 213 nm Nd:YAG nano-second laser
Scan pattern	Line scan
Spot diameter	250 μm
Laser power	90%
Scan speed	50 μm s^− 1^
Laser Fluence	2.6 J cm^− 2^
Repetition rate	20 Hz
Carrier gas flow	0.9 L min^− 1^ He

For quantitative measurements of the filter disks, samples were prepared as described in Section 4. Only one line-scan (conditions see Table 2) across each filter was performed in a way that the centre of the disk was met. As opposed to the mapping protocol described above, no gold-layer was applied on the samples. Readout intervals in the iTEVA software (Thermo Scientific, USA) were set to 4 s for better signal-to-noise ratio (in the mapping context, a time-resolution of 2.5 s was required to provide a sufficient number of data points per ablation pass to allow for automated data treatment). Integration of the time-resolved data was done via the corresponding tool in the iTEVA software.

### 4 Dried-droplet sample preparation

Circular disks of 5 mm diameter were cut from ash-free filter paper (Whatman 589/1, black ribbon, ashless, 110 mm filter diameter) using a steel punching tool. These pre-cut disks were applied on glass microscopic slides using double-sided tape, as proposed in [9]. On these targets, liquid samples were dispensed using an Eppendorf pipet and slowly dried. Samples prepared in the analytical laboratory (i.e., standards, reference material) were dried under ambient conditions in a VFT 1525 ultraclean laminar flow hood (WEISS Technik, Austria). Samples prepared under realistic conditions (method cross-validation, see Section 3) were dried directly in the laboratory, only covered with a tilted glass petri-dish to avoid apparent contamination. The procedural blank of both approaches was monitored and no significant contribution to the analyte signal was observed.

### 5 Conventional ICP-OES procedure

For conventional analysis via pneumatic nebulisation (PN), the samples were diluted 20-fold with 1% (v/v) HNO_3_. Indium was added as internal standard (final concentration: 1.5 μg mL^− 1^) and a conventional Meinhard-type glass nebulizer mounted onto a glass cyclonic spray chamber (both: Thermo, USA) was used for sample introduction. A CETAC ASX-520 autosampler (CETAC Technologies, USA) was used for automated sample uptake, while a peristaltic pump assured long-term stability. Initial method development was achieved by optimizing signals in terms of signal-to-noise ratio. Signals were recorded during PN of a diluted and spiked sample solution (see Table 1). Performance of the system (peak position) was checked prior to each measurement session with a spiked and diluted sample solution. Background-corrected emission signals (integration time: 6 s, n = 4) were recorded and processed using iTEVA software (Thermo Scientific, USA). Quantification of phosphorus was done via external aqueous calibration and normalization to the respective indium signal.

### 6 Fermentation process

The fermentation samples analyzed in this contribution were taken from a chemostat fermentation process (continuous feed of fresh nutrient medium and continuous harvest of the culture broth). The objective of the biochemical investigation was to optimize conditions for biological methanogenesis. This process aims at biochemically reducing carbon dioxide to methane gas in the presence of hydrogen. Similar to established chemical methane synthesis following the Sabatier process [10], biological methanogenesis will allow converting hydrogen or a mixture of hydrogen and carbon monoxide (syngas) into methane gas. Excess energy produced, e.g., in power plants using alternative/renewable energy can thus be effectively converted to methane gas. As opposed to hydrogen, methane can be safely stored and easily distributed via existing gas networks. Compared to the Sabatier process, biological methanogenesis can be achieved at lower temperatures and is more tolerant towards primary gas purity, resulting in a better process efficiency. Based on previous reports of biological methanogenesis [11], [12], limitations in phosphorus concentration were studied here, also with the aim of optimizing future media composition. The process medium contained typical sources of nitrogen, minor concentrations of inorganic salts and typical trace elements. Carbon dioxide was used as exclusive carbon source for the process.

The harvested process medium was initially stored at − 18 °C. Upon arrival in the analytical lab, the samples were unfrozen (at 2 °C), centrifuged (3000 rpm, 5 min, Hettich-EBA 20, Germany) and the supernatant clear solution was stored at 2 °C until further analysis. Hence, the total dissolved phosphorus fraction (TDP) was considered for quantification. As any suspended matter was removed during the centrifugation step, acidification of samples was sufficient for conventional nebulizer-based ICP-OES measurements.

## 3 Results & discussion

### 1 Reproducible sample preparation and representative sampling

In most reports on dried-droplet sample preparation in combination with laser ablation, the smallest possible dried sample residues were sought for [13], [14], [15], [16], [17], [18], [19], [20], [21], [22]. Small spots can be ablated completely with only a few laser shots. This is advantageous, since it provides good sensitivity, comparable to conventional sample introduction systems [15]. This way of droplet preparation requires the use of a hydrophobic surface which allows the droplet to continuously shrink while drying (in all reported cases, a polymeric surface).

**Fig. 1:**
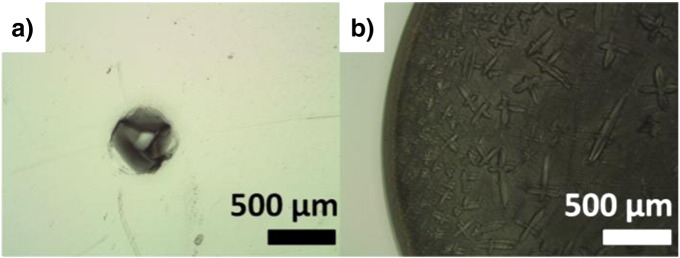
a) 5 μL of aqueous standard (200 μg mL^− 1^ P), and b) 5 μL of fermentation sample, both dried on polyethylene substrate (dried residue in b) only partially visible).

Following these reports, we applied biochemical fermentation media onto polyethylene disks. Different dilutions (1:1, 1:2, 1:5, 1:10, all diluted with water) were prepared. The obtained dry residues were different in shape and size. Especially, dried residues of aqueous standard solutions had a significantly different geometry than those of the fermentation broth (see Fig. 1). This might be due to foam-suppressing agents used during fermentation or due to differences in salt concentration. In any case, these differences require the addition of an internal standard. As a consequence of adding an internal standard, sample handling would become cumbersome and would not be possible on location as outlined earlier.

Recently, Aramendía et al. showed an alternative way for dried-droplet sample preparation [9]. They prepared pre-cut disks of filter paper, which were attached to a hydrophobic surface. When applying liquid samples onto these pre-cut filter-disks, the liquid is trapped on the confined area which is defined by the paper disk. After drying, each of these spots has exactly the same geometry which facilitates sample localisation and measurement. To compensate for the loss in sensitivity (the sample does not contract to a small spot any more), it is possible to apply comparatively large volumes on the filter disks (300 μL on a 16 mm diameter disk [9]).

**Fig. 2:**
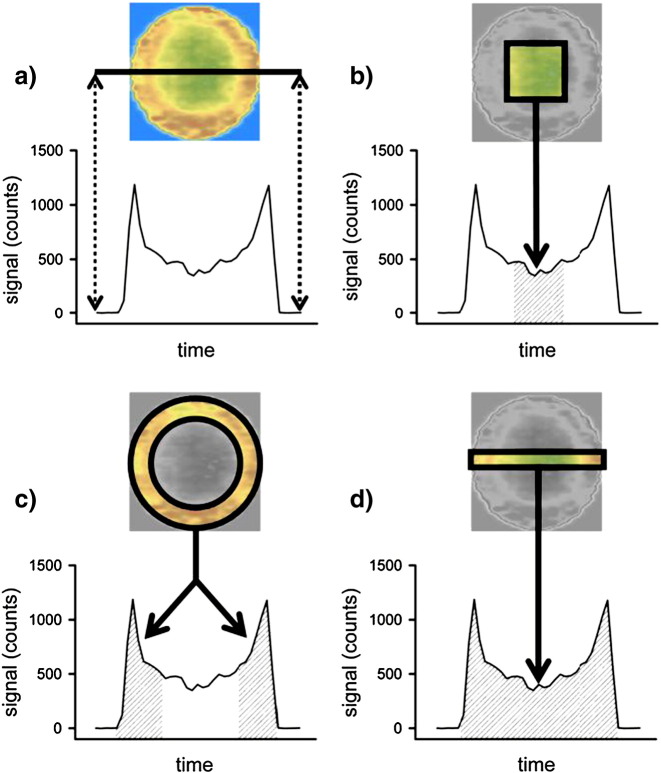
a) The phosphorus distribution within the droplet is not laterally homogeneous (blue: low signal, brown: high signal). A radial line-scan yields a U-shaped response. Results according to previously reported quantification approaches b) [9] and c) [23], as well as according to the proposed representative approach d) (this publication) are schematically shown. The highlighted (projected) area is used for quantification in the respective quantification approaches.

The procedure described in [9] yields dried spots of uniform and highly reproducible geometry. Although the macroscopic sample geometry is controlled when using this approach, the lateral distribution within the filter disk is not homogeneous. To exemplify this effect, Fig. 2 a) shows the lateral distribution of phosphorus on a pre-cut filter disk, prepared and measured as described in 3, 4 (liquid sample: spiked fermentation broth). The phosphorus signal changes substantially within the dried filter disk. The extent of this local pre-concentration depends on the matrix of the liquid sample; for example, when analyzing the area in the centre of the paper filter disk, Aramendía et al. report 20-30% lower signals in purely aqueous solutions compared to spiked sample solutions [9]. This situation is not satisfactory as it calls for the use of an internal standard, standard addition, isotope dilution, or matrix-adjusted calibration.

Taking a closer look at existing measurement strategies for pre-cut filter disks in the context of dried-droplet analysis, only two approaches have been reported so far. Either, a square sub-sample was ablated in the centre of the disk [9] or a ring-shaped sub-sample was analyzed [23]. Obviously, the first approach would under-estimate the concentration in the case depicted in Fig. 2 b) because phosphorus is predominantly present in the rim of the paper disk and thus not included in the measurement. Contrarily, the second approach would over-estimate the concentration in the present samples (see Fig. 2 c)). To summarize, both approaches are systematically biased since non-representative sub-samples of the paper disks are analyzed. One possible way to completely circumvent problems related to the centro-symmetrical inhomogeneity would be to ablate the entire sample as in the case of preparing samples on hydrophobic surfaces. However, considering the size of the filter disks (several mm) and the usually available laser-spot diameters (of a few hundred μm at maximum) in combination with typical repetition rates in the Hz-range, complete ablation of the filter disk with nanosecond laser systems would result in a very time-consuming approach.

Therefore, we suggest applying an alternative measurement protocol, which combines the advantages of preparing samples on pre-cut filter disks with the advantages of ablating only a fraction of the entire filter area. However, and in contrast to existing approaches, this fraction is selected such that it forms a representative sub-sample of the initially liquid droplet. We propose performing line-scans that pass over the entire circular disk. The signal obtained is then integrated which results in a representative sub-sample (see Fig. 2 d)). This hypothesis is based on the assumption that any chromatographic migration effects on a circular area will lead to centro-symmetrical patterns. Hence, the radial sampling approach should be representative for the deposited liquid droplet. However, only the integrated signal will be representative for the liquid sample as the signal will fluctuate along the ablation path due to chromatographic patterns (see the U-shaped response in Fig. 2). The viability of this hypothesis will be further exemplified in the following paragraphs. For the sake of completeness, it has to be noted that radial line-scans have been previously reported for the analysis of dried sample droplets [24]. However, the approach reported by Kumtabtim et al. still required the application of matrix-matched calibration because sample geometry was not controlled (freely dried droplets were investigated). Combining paper filter disks with the proposed radial measurement protocol therefore provides new insights in dried-droplet quantification.

### 2 Figures of merit

**Fig. 3:**
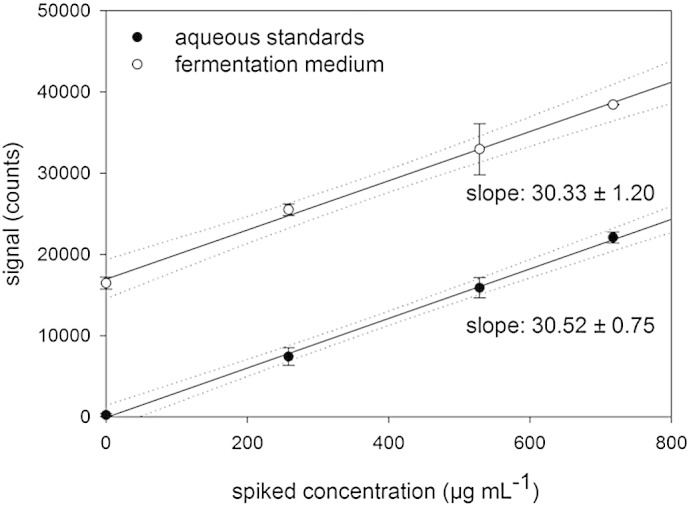
Slopes of standard addition (spiked fermentation medium) and aqueous calibration lines (data points: n = 4, error bar = 1 standard deviation, uncertainties in slope: standard error of the linear regression).

In order to verify the hypothesis that integrated radial line-scans indeed yield representative information, the slopes of aqueous calibration and standard addition were compared. To this end, a representative sample of biochemical medium was spiked with increasing concentrations of phosphorus, corresponding to the concentration levels applied in aqueous calibration. As can be seen in Fig. 3, a good agreement between the slopes of the two curves was obtained. The similarity in slope is a first indication for the absence of differences in signal generation related to sample matrix.

In a second step, the phosphorus signal in the presence of three model substances which are likely to be co-present in the fermentation samples was studied. For that purpose, aqueous solutions containing 500 μg mL^− 1^ P were prepared with incrementally increasing concentrations of sodium chloride, saccharose and tartaric acid (0.3, 1, 3, 10, 30, and 100 g L^− 1^). The latter two compounds are model substances for organic matrix in general. The blank signal of all three substances was checked and found to be ≤ blank level. The results obtained were compared with a purely aqueous solution of the same phosphorus concentration to obtain recoveries in presence of the model matrix. Recoveries within 90%-110% were considered as tolerable (given the reproducibility of laser measurements, see Table 3). Concentrations of up to 100 g L^− 1^ NaCl, 30 g L^− 1^ saccharose, and 10 g L^− 1^ tartaric acid were found to be tolerable. In real fermentation broth samples, Na concentrations in the range of 11 g L^− 1^ – 16 g L^− 1^ were found (determined via dilution followed by PN sample introduction to ICP-OES).

**Table 3:** Figures of merit.

	Laser	Nebulizer
Detection limit (LOD)^a^	10 μg mL^− 1^	0.3 μg mL^− 1^
Quantification limit (LOQ)^a^	40 μg mL^− 1^	1 μg mL^− 1^
Linearity tested until	3,500 μg mL^− 1^	24 μg mL^− 1^
Reproducibility (n = 4)	10% RSD (5.3% - 14%)^b^	1.5% RSD (1.1% - 2.4%)^b^

a in the undiluted sample.

b at different concentration levels, ranging from 10xLOQ to the highest standard level used for linearity check (average, in brackets: minimum and maximum).

To further investigate the effect of sample load, the sample volume applied on the pre-cut filter paper was incrementally increased. The ratio signal/volume is constant up to 17.5 μL of applied sample (see Fig. S1, Appendix A). Also in this investigation, increased sample load had no observable effect on the analytical performance.

The applied sample volume directly affects the achievable sensitivity, and therefore, larger sample volumes would be beneficial in terms of detection limits. With aqueous standards, a maximum capacity of 1.3 μL mm^− 2^ could be applied (25 μL sample dispensed on a disk of 5 mm diameter and 19 mm^2^ area which is comparable to the result reported by Aramendía et al. [9]). However, only 0.9 μL mm^− 2^ of the fermentation broth could be applied in a straightforward way (i.e., 17.5 μL dispensed on a filter of 5 mm diameter). Volumes above 17.5 μL often tended to leave the pre-cut filter disks, probably due to foam-suppressing agents added during fermentation. Hence, to avoid any spilling, a volume of 10 μL (corresponding to 0.5 μL mm^− 2^) was used for all samples and standards.

The linearity of the phosphorus response was checked by analyzing spiked fermentation medium samples up to 3.5 g L^− 1^ P (expected concentrations in the samples are below 2.5 g L^− 1^ P).

From aqueous calibration curves, the detection (3 s) and quantification (10s) limits presented in Table 3 were obtained. In comparison to conventional ICP-OES techniques, the sensitivity is 40 times lower. However, considering the minimum sample preparation required, the absence of an internal standard, as well as the possibility for sample storage, we consider the method as fit-for-purpose in the given analytical context.

To further test the trueness of the method proposed, a certified reference material (IAEA-153, Trace Elements in Milk Powder) was analyzed by LA of dried droplets prepared as described above. The reference material was quantified via external calibration using aqueous P-standard solutions. The experimental result for the phosphorus concentration of 10.1 ± 0.7 mg g^− 1^ (± 1 standard deviation) agrees well with the certified value of 10.1 mg g^− 1^ (95% confidence interval between 9.0 mg g^− 1^ and 11.0 mg g^− 1^). The milk powder was reconstituted with water to obtain a solution of 250 μg mL^− 1^ phosphorus, which is similar in concentration to the biochemical media.

Although the matrix of the reference material is different from that of the samples investigated, we consider the correct quantification of the reference material as additional validation, accompanying the abovementioned experiments regarding standard addition and matrix tolerance. To our knowledge, there is no certified reference material with a suitable matrix composition for the analytical question at hand.

### 3 Application example

In order to assess the trueness of the method, a set of 20 different fermentation samples with P-concentrations ranging from 200 μg mL^− 1^ to 1,800 μg mL^− 1^ was analyzed by the laser ablation method. To this end, 10 μL of the samples were applied on pre-cut paper filter disks, dried and ablated using the proposed radial line scan. Signals were integrated over the entire filter (n = 2 filters per sample) and quantified versus aqueous calibration standards also applied on filter paper disks.

**Fig. 4:**
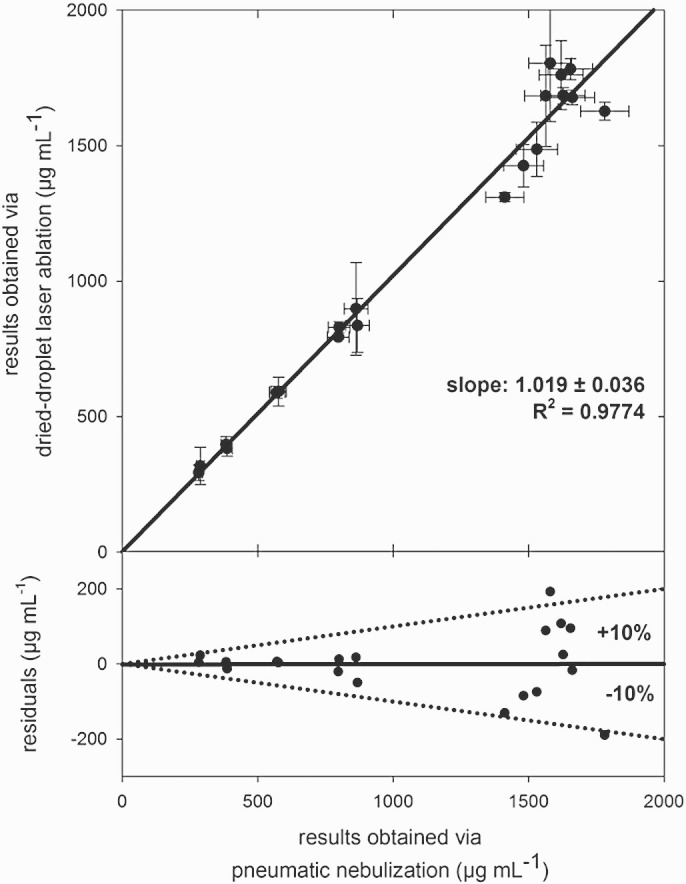
Correlation between results obtained via laser ablation-ICP-OES analysis of dried droplets (n = 2) and via conventional liquid pneumatic nebulisation ICP-OES analysis (n = 4), respectively. Error bars correspond to 1 standard deviation, uncertainty of slope: standard error of the linear regression.

The same set of 20 samples was also diluted as described in Section 5 and analyzed using the conventional nebulizer/spray-chamber set-up in combination with ICP-OES. Between the results obtained with both methods, a good correlation was obtained and residuals from the interpolated line are in most cases below 10%, across the entire range of concentrations (see Fig. 4). Considering the potentially high biochemical variability (reproducibility of sampling and sub-sampling), the measurement variability observed is satisfactory in the context investigated.

## 4 Conclusion

In this contribution, we present a method for analyzing challenging liquids, based on laser ablation of dried sample droplets deposited on pre-cut paper filter disks. Our method solves the problem of non-representative sampling, which so far necessitated internal standardisation, matrix-adjusted calibration, standard addition, or isotope dilution. With the approach presented, straightforward external aqueous calibration becomes possible. Key feature of our method is to perform radial line-scans across the entire dried droplet and to integrate the resulting transient signal. Reproducibility and detection limits of the approach presented are higher (10-fold and 40-fold, respectively) than those of existing routine techniques relying on nebulisation of diluted samples. However, the present method offers significant improvements in terms of facilitated sample preparation, storage and calibration, which outweigh the limitations mentioned. Moreover, issues related to sensitivity could be easily overcome with an alternative detection set-up such as LA-ICP-MS or laser-induced breakdown spectroscopy (LIBS). For upcoming research, the method will be extended to allow for simultaneous multi-element quantification in biochemical media, also in combination with automated sample collection and preparation. Thus, it will be possible to follow the fermentation process with superior time-resolution. The general viability of the proposed measurement protocol will be investigated, also with other matrices, such as process additives (silicon oil, anti-foam agents) and blood.
