# Supplementary material for: Radial line-scans as representative sampling strategy in dried-droplet laser ablation of liquid samples deposited on pre-cut filter paper disks
Source: Spectrochim Acta Part B At Spectrosc. 2014 Nov 1;101:123–9. doi: 10.1016/j.sab.2014.07.023 (PMC4210661; doi:10.1016/j.sab.2014.07.023)
Supplement: Supplementary file 1 — Supplementary material [file mmc1.pdf]

Appendix A. Supplementary data

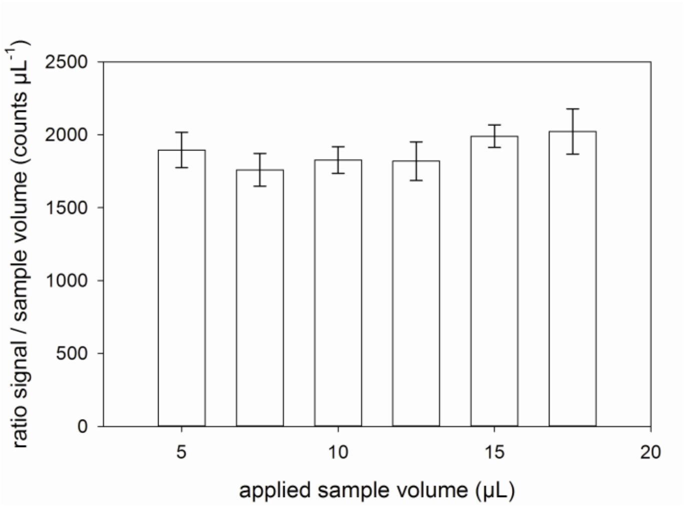

Figure S1. Relative signal intensity as a function of the sample volume applied (n = 4).
